# A simple technique for assessing the cuticular diffusion of humic acid biostimulants

**DOI:** 10.1186/s13007-019-0469-x

**Published:** 2019-07-31

**Authors:** Marcela Smilkova, Jiri Smilek, Michal Kalina, Martina Klucakova, Miloslav Pekar, Petr Sedlacek

**Affiliations:** 1 Institute of Physical and Applied Chemistry, Brno University of Technology, Faculty of Chemistry, Purkynova 464/118, 612 00 Brno, Czech Republic; 2 Materials Research Centre, Brno University of Technology, Faculty of Chemistry, Purkynova 464/118, 612 00 Brno, Czech Republic

**Keywords:** Diffusion, Hydrogel, *Prunus laurocerasus*, Liquid fertilization, Humic substances, Plant cuticle

## Abstract

**Background:**

Experimental determination of the extent and rate of transport of liquid humates supplied to plants is critical in testing physiological effects of such biostimulants which are often supplied as foliar sprays. Therefore, an original experimental method for the qualitative investigation and quantitative description of the penetration of humates through plant cuticles is proposed, tested, and evaluated.

**Results:**

The proposed method involves the isolation of model plant leaf cuticles and the subsequent in vitro evaluation of cuticular humate transport. The employed novel methodology is based on a simple diffusion couple arrangement involving continuous spectrophotometric determination of the amount of penetrated humate in a hydrogel diffusion medium. *Prunus laurocerasus* leaf cuticles were isolated by chemical and enzymatic treatment and the rate of cuticular penetration of a commercial humate (lignohumate) was estimated over time in quantitative and qualitative terms. Different rates of lignohumate transport were determined for abaxial and adaxial leaf cuticles also in relation to the different cuticular extraction methods tested.

**Conclusions:**

The proposed methodology represents a simple and cheap experimental tool for the study on the trans-cuticular penetration of humic-based biostimulants.

## Background

Foliar application of fertilizers and biostimulants has become a popular method in the field of agronomy and the plant nutrition since its first use in the early twentieth century. Nowadays, foliar uptake of nutrients widely complements standard root fertilizer treatments [1, 2]. Most aerial plant organs (leaves, stem, etc.) are long-known to be able to take up nutrients from sprays [3, 4]. Transcuticular penetration into leaf tissues and sorption on the leaf surface plays a key role in the foliar application of nutrients [5–7], surfactants [8, 9] and different types of pesticides [10–15].

Bidirectional transport of diverse substances in and out of plants is controlled primarily by the plant cuticle [16], a membrane which covers the aerial plant parts and forms the natural interface between plant organs and a surrounding environment [17]. From a chemical point of view, it can be considered as a heterogeneous composite material which is formed by lipophilic components such as waxes, water insoluble polymers cutin and/or cutan and phenolic compounds like flavonoids, mixed together with hydrophilic polar compounds such as polysaccharides [18].

The cuticle plays its biological role principally as a barrier to control the movement of gases, water and solutes and to impart pathogen resistance [17, 19–21]. Furthermore, it protects a plant against abiotic factors such as rain, frost and ultraviolet light [21, 22], and also against adverse biotic impacts of insects, pests, mycosis, etc. [20, 23]. Naturally, as the correct functioning of plant cuticles is crucial for the well-balanced uptake of nutrients, minerals, adjuvants, or for plant growth preparation, cuticle-penetration experiments as well as laboratory studies of the structural physico-chemical properties of cuticles are an essential part of plant-nutrition research.

For more than 50 years, scientists have continuously been focusing their experiments on the penetration of active compounds through plant cuticles, or on their adsorption on the cuticle surface [3, 24]. For the purpose of the experimental determination of the extent and rate of foliar absorption of a nutrient, several techniques directly involving intact leaves were tested, e.g. dipping, brushing, sticking, spraying, or the droplet method [3]. However, attention has gradually been paid also to experiments performed with cuticles in an isolated form. The first successful attempts at cuticle isolation were made by Orgell, who developed a method based on the treatment of leaves by pectinase [25]. A chemical alternative to this enzymatic method is isolating cuticles with zinc chloride as introduced by Holloway and Baker [26] and further utilized by Solel and Edgington [10, 27, 28]. Various experimental settings have been used for cuticular permeability trials, such as a sole transpiration chamber [29], a side-by-side transport chamber, in which the cuticle is located between two compartments filled with donor and receptor solution [24], or a tube-in-tube setup, where the cuticle is affixed on the opening of small tube filled with deionized water and submerged in the larger tube with a donor solution [30].

Either in experiments performed with intact plant organs or with those utilizing isolated plant cuticles, another crucial step in the development of a particular methodology for studying foliar absorption is the use of an analytical method for the quantification of the cuticle-permeating compound. Foliar uptake of inorganic compounds has been often studied by means of radioactive isotope methods, where different radioactive isotopes of elements are used e.g. ^14^C [30, 31], ^32^P [32–35], ^42^K or ^45^Ca [36], or ^86^Rb, ^45^Ca, ^36^Cl and ^35^S [37]. Radionuclide assays have also been utilized successfully in foliar absorption studies of organic compounds such as urea [30] and atrazine [11]. Among other methods for studying the foliar uptake of organic molecules, HPLC was used to determine the quantity of the organic dye Congo Red and fungicides on the leaf surface [38]. Another experimental approach is based on tracking the penetrating compounds indirectly by observing their effects. This approach was used, for example, by Solel and Edgington [10] or, more recently, by Zelena and Veverka [13], who studied the rate of the transcuticular movement of fungicides by measuring the propagation of inhibitory zones in agar gel fed with a fungus sensitive to the tested fungicide. The fungicide was always applied on top of the cuticle, placed in the center of the agar-filled Petri dish.

Humic substances are complex organic mixtures that fulfil a range of important functions in ecosystems and that are essential for their proper functioning. They represent an essential fraction of the natural organic matter of soils, peats, and young coals. In a dissolved form, they can also be found in aquatic systems such as rivers and lakes. A growing understanding of the positive effects of the presence of humic substances in their natural habitats has motivated the preparation of commercial products based on isolated natural or artificially synthesized humic substances. Positive impacts of the utilization of humic-based soil amendments on the chemical [39, 40], physical [41, 42], and microbial [43] fitness of soils have been thoroughly documented. Apart from the application of humic-based materials into soil, the foliar application of soluble humates has gradually become a popular way of application as well. This was initiated by numerous reports on the biostimulant effects of humates, namely on the effect on plant growth [44, 45] and nutrient uptake [46], hormone-like [47, 48] and enzyme-promoting effects [49, 50], as well as some effects enhancing photosynthesis and seed-germination [51, 52]. In particular, in greenhouse experiments using cuttings and young olive plants, Fernandez-Escobar found foliar-applied humic substances extracted from leonardite to have an effect on olive growth [53]; the tested leonardite extracts stimulated shoot growth and promoted the accumulation of elements in leaves. Maibodi et al. suggested that foliar application of humic substances might be of benefit with respect to enhancing nutrient uptake, root development, and drought resistance in ryegrass [54]. In addition, Bettoni [55] showed that a combination of foliar and immersion methods represented the most effective way of applying humic substances originating from leonardite, as far as the tested onion bulb yield and quality was concerned.

However, there is raising debate concerning the generally accepted beneficial effects of commercial humates in agriculture. Olk et al. [56] and Rose et al. [57] reviewed information on the benefits of using humic preparations in agriculture and stressed still ambiguous results. Similarly, Lyons and Genc [58] has pointed out that there is a surprising lack of evidence regarding the effectiveness of the on-farm application of humates, the findings concerning their beneficial effects being inconsistent. Among other recommendations, these authors call for a comprehensive physico-chemical characterization of humates and for a careful assessment of the mechanism of their foliar action. Thereby, the experimental determination of the rate of absorption and transport of humate solutions applied to plants also as foliar sprays is critical as preliminary step for assessing their biological effects. Recently, some attempts have been made in describing the root pathway of the humate absorption. For instance, Kulikova studied the uptake of leonardite humic substances by plant root and its transport and spatial distribution among the plant tissues using microautoradiography [59]. Nevertheless, as yet, no experimental procedure has been proposed for a systematic assay of the transcuticular uptake of humates. The aim of this paper is hence to introduce an original in vitro technique as a simple experimental option for this task. The proposed method enables the investigation and quantitative description of the penetration of humates through the plant cuticle via spectrophotometric monitoring of the diffusion of humates through an isolated plant cuticle fixed between a donor and an acceptor agarose hydrogels forming the common diffusion couple arrangement. The basic design of the method follows on from our previous work [60, 61]. The usability of the technique was tested on artificial lignohumate as a model commercial product and on cuticles obtained by enzymatic and chemical means of isolation.

## Materials and methods

### Isolation and characterization of cuticles

Leaf cuticles were isolated by two different methods (chemical and enzymatic) as described previously [10, 25]. In both cases, plant cuticles were isolated from *Prunus laurocerasus* (see “Results” and “Discussion”). Firstly, undamaged, young, fully-expanded leaves were immersed in distilled water. For the every plant leaf, the leaf blade (the lamina) was carefully cut off by scalpel from the other leaf parts (veins, petiole). In the case of enzymatic isolation, the lamina was then immersed into the isolation solution consisting of citric buffer (0.1 M and pH 3.5), supplemented with 2.5 wt% of pectinase from *Aspergillus niger* (> 1 units/mg, Sigma-Aldrich), 2.5 wt% of cellulase from *Trichoderma logibrachiatum* (> 1 units/mg, Sigma-Aldrich) and 0.25 wt% of sodium azide (p.a., Sigma-Aldrich). After 6 weeks, the leaf tissue, including isolated cuticles, was placed in distilled water and degraded mesophyll was gently removed by brushing. The chemical isolation procedure differed only in the composition of the isolation solution, i.e. 60 wt% zinc chloride (≥ 97%, Sigma-Aldrich) dissolved in concentrated hydrochloric acid (35%, Penta), and the shorter time duration of the chemical treatment (3 days).

**Fig. 1:**
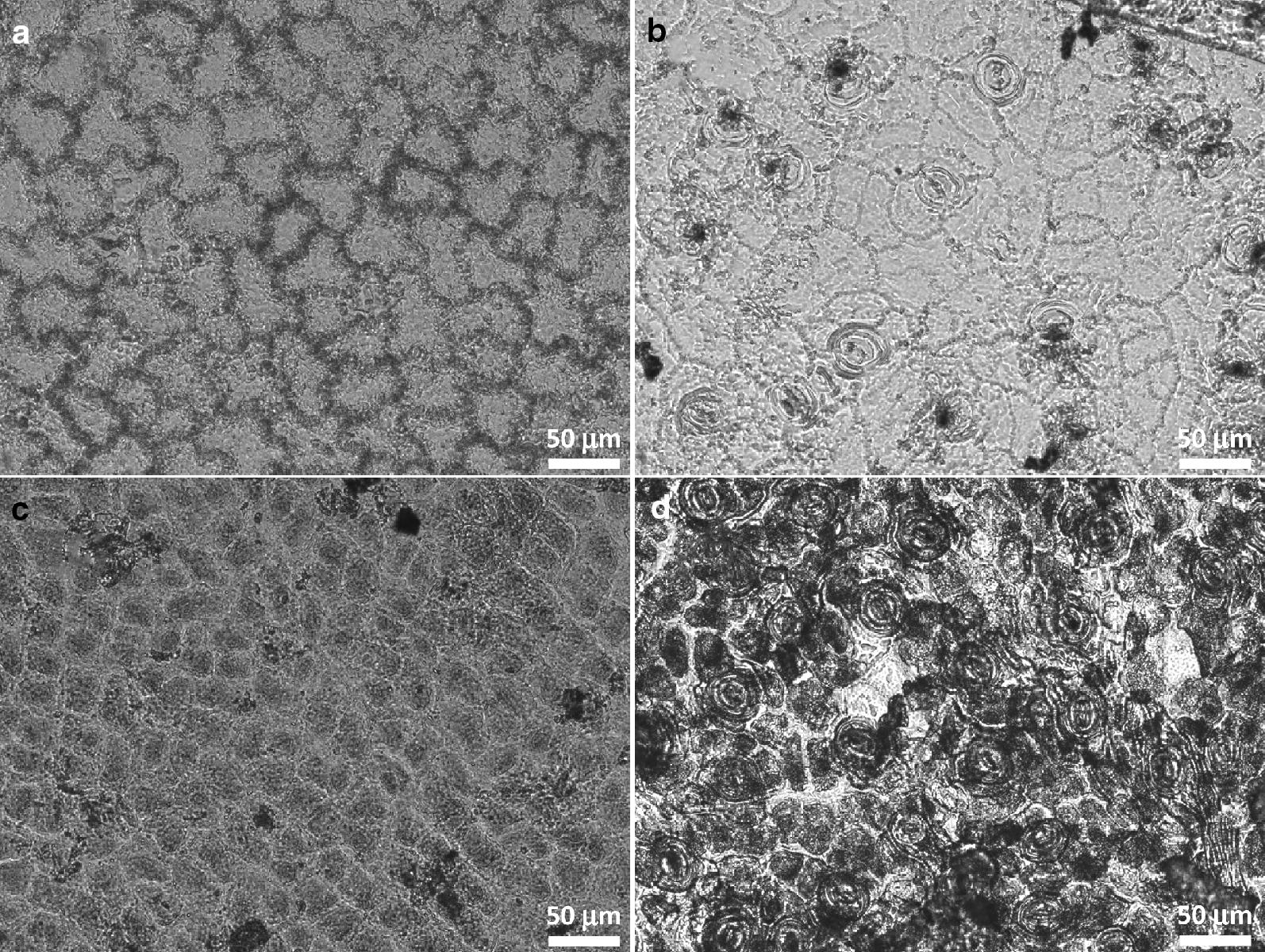
Optical micrographs of the isolated cuticles. Adaxial (**a**, **c**) and abaxial (**b**, **d**) cuticles isolated by chemical (**a**, **b**) and enzymatical (**c**, **d**) methods, respectively

Plant cuticles isolated by both the above mentioned methods were characterized by optical microscopy (Olympus IX71, objective Olympus PLN, magnification 20×, numerical aperture 0.40), by which differences in abaxial (stomatous) and adaxial (astomatous) cuticles were compared and the average radius of stomata was determined (for the optical microscope images of the cuticles, see Fig. 1). Optical microscopy was also used in order to eliminate physically damaged cuticles. The morphology of isolated plant cuticles, especially average cuticle thickness and roughness, was determined by mechanical profilometer (DEKTA kXT, Bruker). VISION 64 was used as the control software and the pressure value of the stylus was set at 3 mg.

### Preparation of hydrogels

All hydrogels used in this work were prepared via the thermoreversible gelation of agarose (< 10% moisture content, Sigma-Aldrich). These hydrogels acted as supporting matrix in which diffusion experiments on the active compound were performed. Lignohumate A was kindly provided by the Amagro (Prague, Czech Republic) and used as model active compound. It represents a commercial mixture of potassium humates and fulvates prepared by hydrolytic-oxidative conversion of technical lignosulfonates under strictly controlled conditions [62, 63]. The method of preparation of hydrogels is described in detail in our previous studies [60, 64]. Donor hydrogels containing 1 wt% agarose with the addition of 1 wt% potassium lignohumate (Lignohumate A) dissolved in distillated water and acceptor hydrogels consisting of only 1 wt% agarose were prepared in PMMA cuvettes (dimensions 10 × 10 × 45 mm). The thermoreversible gelation of agarose took place at ambient temperature and 100% relative humidity for at least 45 min. After the gelation process was completed, excess gel mass above the cuvette edge was cut off by scalpel for all prepared hydrogels. This provided a flat gel surface that allows even contact with the cuticle and with the second hydrogel in the diffusion couple arrangement (see below). This arrangement was obtained in the way that every single isolated plant cuticle was carefully placed between the donor and acceptor hydrogels and the contact area of both cuvettes was isolated from surroundings by parafilm to prevent unfavorable drying.

### Diffusion experiments

**Fig. 2:**
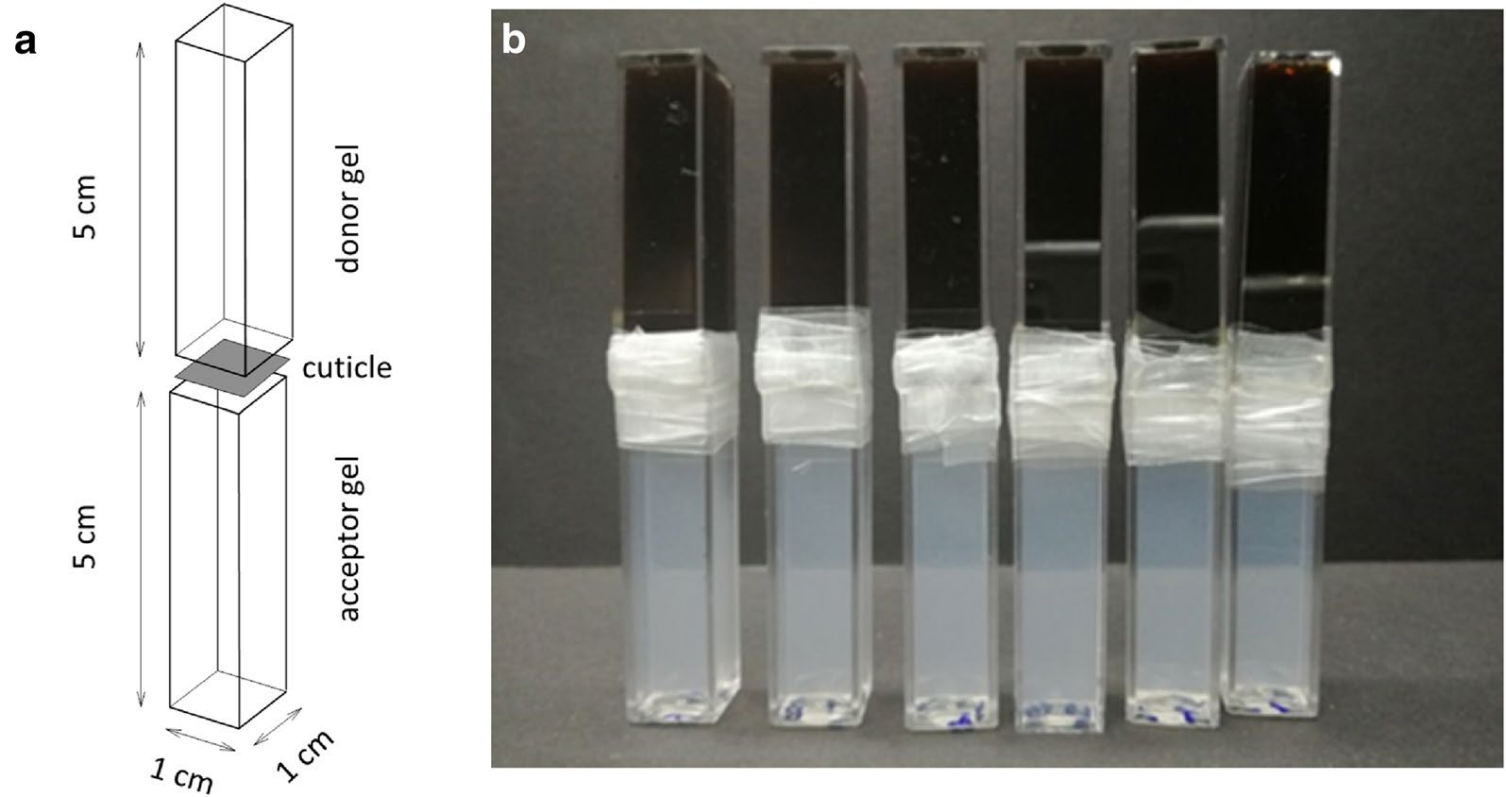
Schematic drawing of the diffusion couple arrangement (individual parts are shifted for clarity) (**a**). Picture of the diffusion couple at the beginning of the experiment (**b**)

Both abaxial and adaxial cuticles isolated by the two different isolation methods (chemical and enzymatic) were subjected to diffusion experiments. As can be seen in Fig. 2, the diffusion couple was formed by two agarose hydrogels—a hydrogel with an homogenously dispersed humate inside (i.e. the donor hydrogel) and a hydrogel with no initial content of the humate (i.e. the acceptor hydrogel)—and the isolated cuticle separating the two gels. As the humate can move freely inside the agarose matrix, it penetrates the cuticle and flows across the concentration gradient from the donor to the acceptor gel. These diffusion experiments were performed with 10 repetitions for abaxial and adaxial cuticles, respectively. During the diffusion experiments, all the diffusion couples were placed in a closed container above water level (to maintain constant humidity of the surroundings). Experimental conditions—in particular, relative humidity (100%) and temperature (25 °C)—were held constant during the whole experimental period.

The transport of lignohumate through the plant cuticle into the acceptor hydrogel was measured by a UV–VIS spectrophotometry (Varian Cary 50). At selected time intervals, the cuvettes were taken out and UV–VIS spectra of lignohumate in acceptor hydrogels were collected at distances from the hydrogel-cuticle interface ranging from 3 to 40 mm (with 1 or 2 mm increment in distance depending on the actual rate of the color change). The measurement of UV–VIS absorbance at different distances from the interface was performed by means of a special in-house made accessory providing controlled fine vertical movement of the cuvette in the spectrophotometer (see [65] for more detail and picture of the used cuvette holder).

## Results

### Isolation and characterization of cuticles

Two methods of cuticle isolation were tested. The main advantage of the enzymatic method is the use of less harmful isolation agents. The procedure is, therefore, more user-friendly and suitable for routine laboratory use. Furthermore, the presence of any structural artifacts arising from chemical damage to the cuticle is less likely than in the case of more drastic chemical treatment in hydrochloric acid. On the other hand, the enzymatic method is significantly more time-consuming in comparison with chemical isolation. In our study, however, both techniques resulted in the isolation of cuticles which were strong and easy to manipulate. Optical microscopy was used in order to exclude mechanically damaged cuticles, to distinguish between abaxial and adaxial cuticles, and to sort them for use in subsequent diffusion experiments.

Furthermore, microscopical observation of the cuticles also revealed some interesting differences in the efficiency of the two isolation methods. Apparently, chemical treatment of the leaves led to the more efficient removal of cell debris as compared to the enzymatic procedure (see the black spots on the micrographs shown in Fig. 1). This finding corresponds well with general differences in cuticle thickness for the two isolation methods as revealed by profilometry, i.e., it was found that the thickness was always greater for enzymatically isolated cuticles (see Table 1). Anyway, both types of cuticles (i.e. chemically and enzymatically isolated ones) were included in the next step of the testing of the proposed methodology, i.e. in the diffusion couple experiments with agarose hydrogels.

**Table 1:** Basic morphological characteristics of the isolated cuticles

	Avg. cuticle thickness (μm)	Avg. dimensions of stomata (μm)
Chemically isolated		
Abaxial	4.4 ± 1.2	14 × 7
Adaxial	5.5 ± 0.3	–
Enzymatically isolated		
Abaxial	6.7 ± 2.0	12 × 5
Adaxial	9.2 ± 0.9	–

### Diffusion of lignohumate through the cuticles

The main experimental core of the proposed diffusion methodology is based on our previous studies [60, 61, 65, 66]. Generally, as far as the experimental study of molecular diffusion is concerned, hydrogels represent a highly beneficial material form. In the gel phase, the diffusion flow of a solute is not disturbed by thermal or mechanical convection such as in liquid solution. Furthermore, a hydrogel sample of precisely defined shape and dimensions can be prepared, which enables correct description of the diffusion flow by quantitative parameters such as diffusion coefficients. In the current experiments, a simple diffusion couple arrangement was used [24, 67].

In order to evaluate this technique, the diffusion of lignohumate—a model artificial humate—was studied using the above-described experimental arrangement. The composition, structure, and physico-chemical properties of lignohumate are discussed in detail elsewhere [63, 68], as well as its biological effects [69, 70]. For our purposes, the main practical benefits of using lignohumate as a model humic biostimulant are its very high water solubility, low molecular size, and reproducible means of preparation, the latter resulting in a stable and standardized structure with standardized properties.

**Fig. 3:**
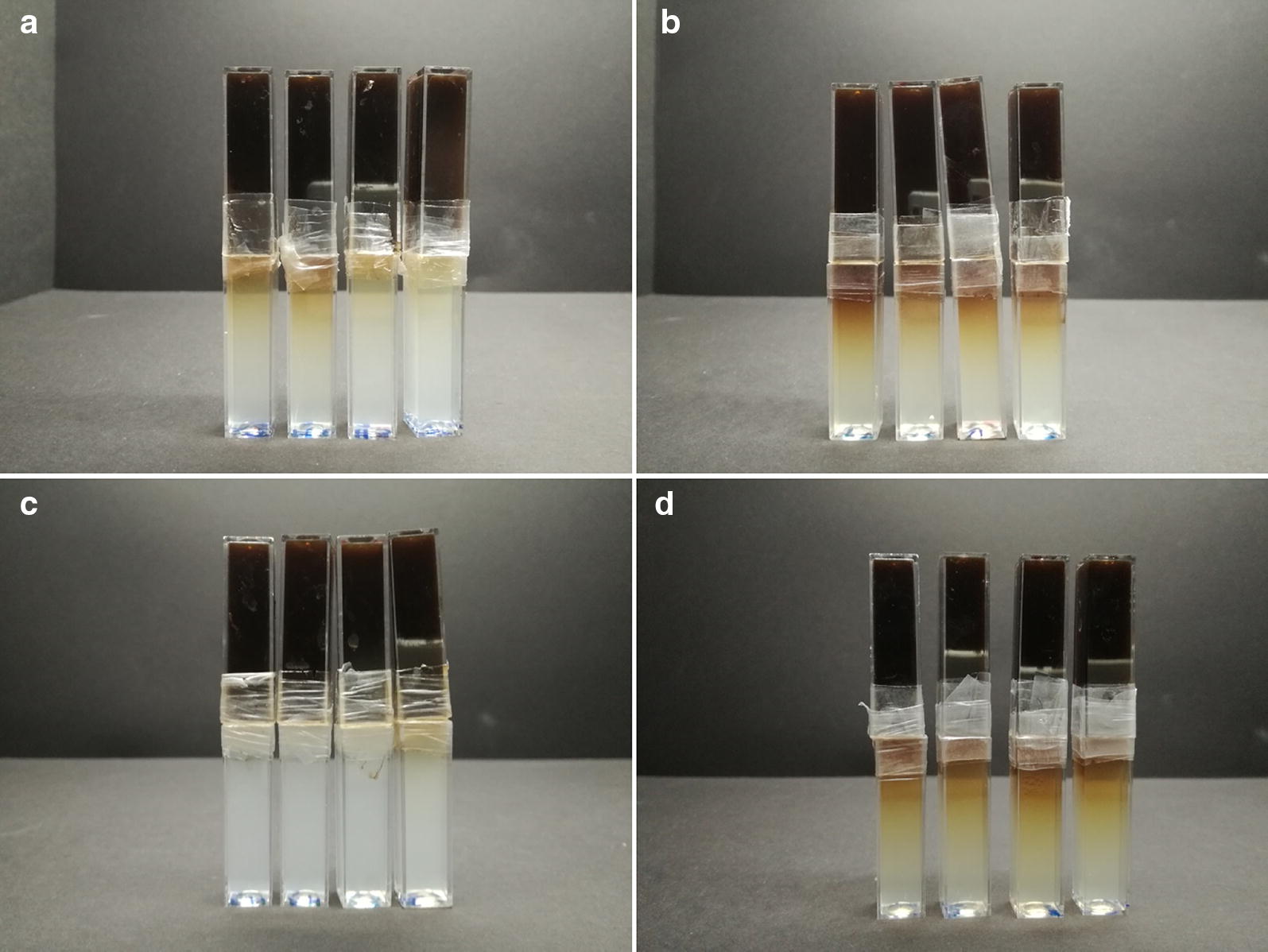
Diffusion couples after 7 days of experiment. Comparison of diffusion progress for adaxial (**a**, **c**) and abaxial (**b**, **d**) cuticles and for chemical (**a**, **b**) and enzymatical (**c**, **d**) methods of cuticle isolation

In Fig. 3, the movement of the dark-brown-colored lignohumate through the cuticle and its subsequent diffusion into the optically transparent agarose gel can be observed visually. It is evident how the local concentration of lignohumate and its depth of penetration increases with time and that the adaxial and abaxial cuticles show large differences in their barrier properties. As expected, the rate of diffusion is higher in the case of abaxial cuticles. The explanation is straightforward; isolation of stomateous cuticles results in membranes with freely permeably holes of several microns in size (guard cells which protect stomata against penetration are lost during the isolation). The penetration of lignohumate through these membranes is therefore controlled by the size and density of the holes and by the rate of free diffusion of lignohumate in solution rather than by the barrier properties of the neighbouring lipophilic cuticle area.

In addition, visual evaluation of the concentration of lignohumate in acceptor gels indicates a higher diffusion rate in the case of chemically isolated abaxial cuticles as compared to enzymatically isolated ones. For the adaxial cuticles, the differences were less pronounced. This can be explained by the already mentioned outcomes of the structural analysis of the cuticles. Enzymatically isolated cuticles are thicker and mainly exhibit higher levels of contamination by debris from the leaf. It is likely that in the case of enzymatically isolated cuticles stomata are partially blocked by leaf debris and, consequently, contribute less effectively to the transport of lignohumate.

### Quantitative description of the transcuticular transfer of lignohumate

**Fig. 4:**
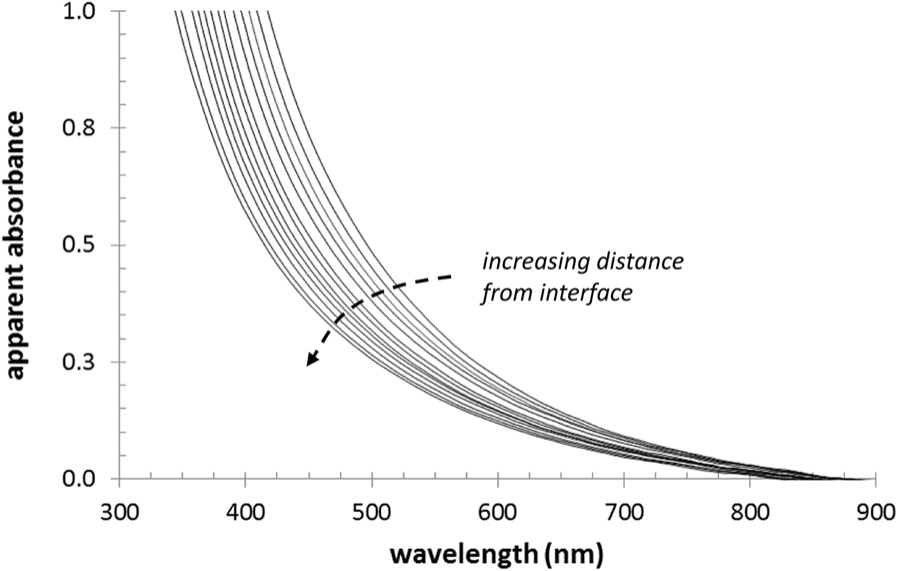
UV–VIS spectra measured at different positions in the acceptor gel (adaxial chemically isolated cuticle, after 96 h). Spectra were taken in 2 mm increments of distance from the cuticle

**Fig. 5:**
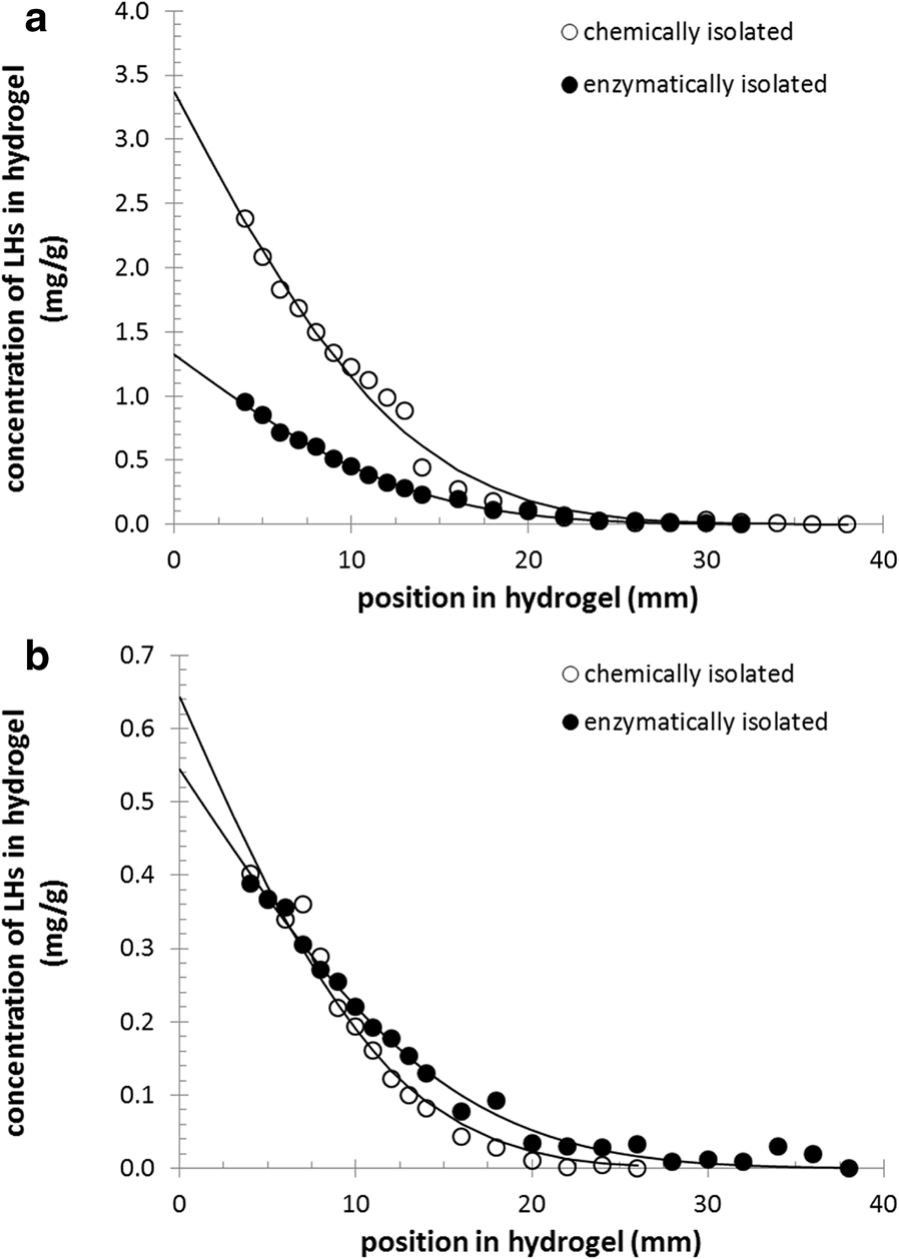
Concentration profiles of lignohumate in the acceptor gel of the diffusion couple **a** after 96 h of diffusion through abaxial, and **b** after 168 h of diffusion through adaxial cuticles

Figure 4 shows example of the set of UV–VIS spectra collected at various positions in acceptor hydrogel that illustrates uneven distribution of lignohumate in the gel. It should be noted that each recorded spectrum (shown as the apparent absorbance vs. wavelength) is actually formed by the combination of two separate contributions—the turbidity of the hydrogel caused by light scattering on the solid agarose matrix and light absorption by the dissolved humate in the aqueous phase of the gel. While the former contribution was homogenous and constant for all the measured gels (i.e. it changed neither with time nor with location in the gel), the latter depended on the actual concentration of the humate at a particular time and at a particular point in the gel. In order to calculate the concentration of lignohumate from the respective UV–VIS spectrum, we also measured the spectra of reference samples of agarose gels in which the exact concentration of homogenously distributed lignohumate was achieved by dispersion of a known amount of lignohumate in the agarose solution before its gelation. It is also evident from Fig. 4 that lignohumate provides a continuous absorption spectrum covering a wide range of wavelengths instead of any separate absorption peak. This is a spectroscopic feature typical of humic substances and is the result of their complex structural nature. Therefore, we used a multiple calibration approach in which the concentration of the lignohumate was determined as an average of the values calculated for three different wavelengths (600, 700 and 800 nm). In this way, the concentration profiles of lignohumate in the particular acceptor gels were determined (see Fig. 5).

As can be seen from Fig. 5, the concentration profiles confirmed all above mentioned visual observations. The concentration of lignohumate at a given distance in the acceptor gel was significantly higher when the lignohumate penetrated through an abaxial cuticle. While enzymatically isolated abaxial cuticles had lower penetration rates than chemically isolated ones, no such significant difference was found for adaxial cuticles. The concentration profiles were also subjected to further mathematical processing in order to calculate the total amounts of lignohumate accumulated in the acceptor gels. For this purpose, we fitted the experimentally derived concentration profiles using the following relation

1 $$ c = {\text{A}} \cdot {\text{erfc}}\left( {\frac{x}{\text{B}}} \right) $$

where *c* is the concentration of lignohumate in mg_LH_/g_gel_ at distance *x* from the gel-cuticle interface, and A and B are the fitting parameters. The complementary error function erfc is a non-elementary function of sigmoid shape that generally occurs in non-stationary diffusion equations [71]. The fitting of experimental data was performed by non-linear least square regression using the Solver tool in Excel (Microsoft). With the known fitting parameters A and B at given time *t*, the total diffusion flux of lignohumate across unit area *n* (in g_LH_/m^2^) can easily be determined from the integration of Eq. (1) in the range $x = 0$ to $x = \infty$, which leads to the relation

2 $$ n\left( t \right) = \rho_{\text{gel}} \cdot \frac{{{\text{A}} \cdot {\text{B}}}}{\sqrt \uppi } $$

where the density of the gel ($\rho_{\text{gel}}$) was substituted by the density of pure water for simplicity because of the very low dry matter content of the gels (approximately 1 wt%).

**Fig. 6:**
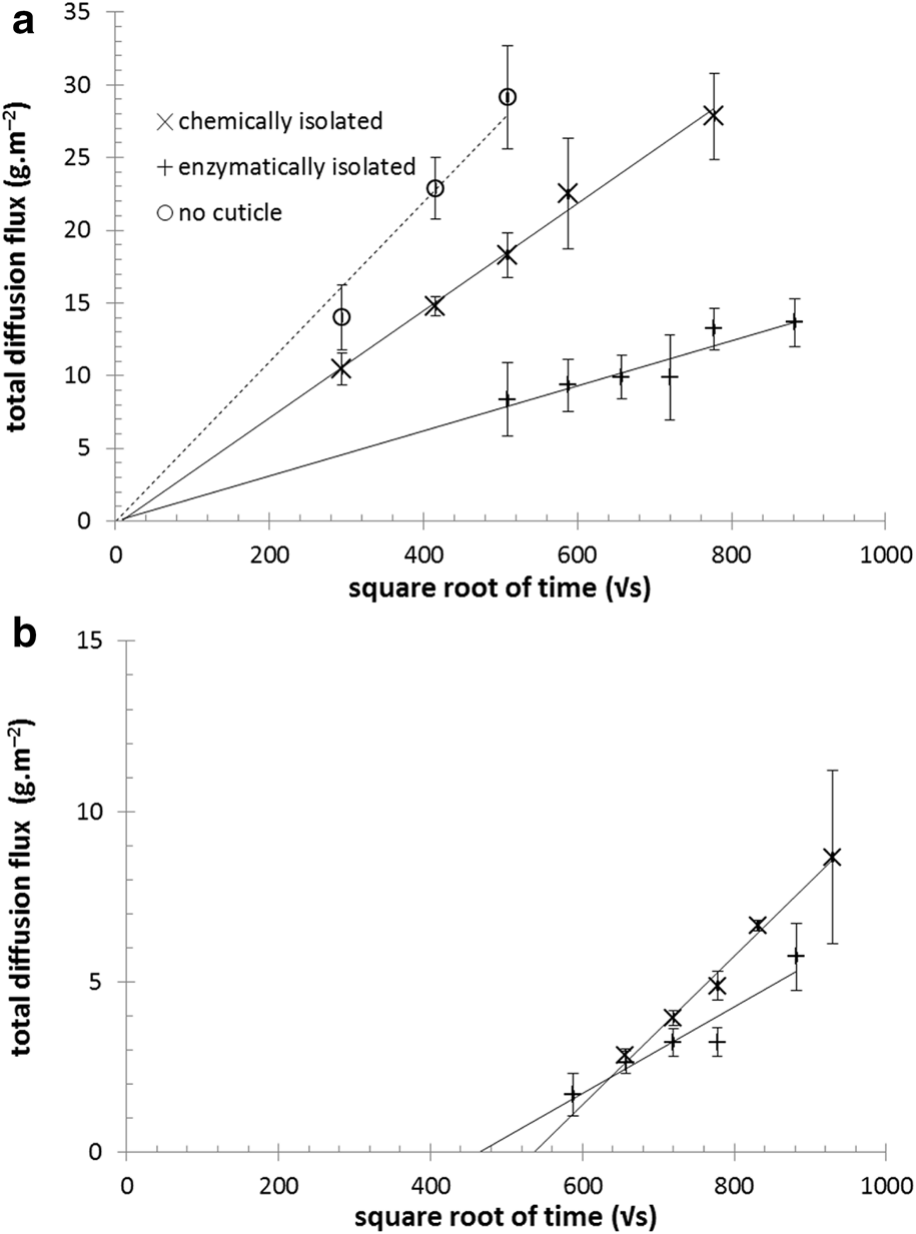
Confirmation of the Fickian type of diffusion process. Dependence of total diffusion flux on the square root of time for abaxial (**a**) and adaxial (**b**) cuticles

For the non-stationary Fickian diffusion of a solute in a composite medium, the total diffusion flux *n* increases linearly with the square root of time. As can be seen in Fig. 6, the linearity of this dependency was confirmed for the diffusion of lignohumate through all forms of the tested cuticles (for the comparison, the results of lignohumate diffusion in absence of any cuticle is shown in Fig. 6a as well). In the case of abaxial cuticles, the linear regression of the function $n = f\left( {\sqrt t } \right)$ crosses the origin of coordinates. In other words, lignohumate penetrates abaxial cuticles instantaneously. This confirms that the stomata on the abaxial side of the leaf, after the removal of the guard cells during the isolation process, represent freely penetrable parts of the cuticle. In contrast, the *x*-axis intercept of the function $n = f\left( {\sqrt t } \right)$ for adaxial cuticles is shifted to significantly higher times. From the intercept, the time needed for lignohumate to penetrate the cuticle (usually called the lag time) was calculated. For both types of adaxial cuticle, quite high lag times were determined. This confirms that in the case of adaxial cuticles molecular transport takes place over a much more tortuous pathway. It is also further evidence that neither of the isolation procedures led to significant mechanical damage to the cuticles in the form of cracks or ruptures.

It was found that while lignohumate needed about 60 h to penetrate enzymatically isolated adaxial cuticles, this lag time increased to about 80 h in the case of chemically isolated ones. Interestingly, after the penetration of the cuticle was complete, the trend was reversed—the amount of lignohumate transported into the acceptor gel increased more rapidly in the case of chemically isolated cuticles compared to enzymatically isolated ones. It is not possible to propose a reasonable explanation for this phenomenon just from the basic structural analysis of cuticles performed in this work, a comprehensive chemical assay of the isolated cuticles would be necessary for a detail discussion of these results (see “Discussion” section).

## Discussion

The urgent need for an assembly of methods for a systematic study of foliar action of humates was claimed recently [58]. To contribute to this complex task, we hereby propose a simple experimental method for a quantitative description of permeability of plant cuticles for liquid humates. The main aim of the present work was to devise and to verify the usability of the proposed methodology.

We are well aware that the proposed experimental approach suffers several general limitations. First of all, the method is based on isolated plant cuticles. Taking into account that isolation of large-sized cuticular membranes is only successful with few species and that the resulting isolates may differ significantly in their structure and chemical composition [72], even the choice of suitable plant becomes a non-trivial issue. In the current work, we proposed and tested *Prunus laurocerasus* as a source plant. The selected plant may not have any direct agricultural relevance as far as the foliar application of humates is concerned. Rather, its choice was based on specific experimental demands with respect to the universality and reproducibility of the developed methodology. The main requirements were as follows: there had to be adequate availability of the plant (both seasonal and regional); the method of cuticle separation had to be simple and reproducible; and the isolated cuticles had to exhibit suitable mechanical properties. From this viewpoint, *Prunus Laurocerasus* was chosen as a suitable candidate. Nevertheless, a proposal of an alternative plant with a specific relevance in current agricultural use of humic-based foliar formulations is highly welcome.

Another issue, which must be considered, is represented by possible artifacts brought by the process of cuticle isolation. Results of optical microscopy and profilometry confirmed higher efficiency of chemical removal of cell debris as compared to the enzymatic treatment. It can be expected that the presence of residual cell debris on the cuticle surface will negatively influence the accuracy of any experiments mapping barrier properties. From this point of view, the results of the structural characterization of the obtained cuticles support the choice of chemical method of isolation. On the other hand, it is likely that more adverse conditions employed during the chemical isolation treatment will result in more severe alteration of chemical composition of the isolated cuticles. It was suggested by several researchers [4, 73], that the isolation can induce changes in chemical structure of the cuticular membrane which may lead to the results of permeability studies different from those performed with intact leaves. Also our results (different permeability of chemically and enzymatically isolated) are consistent with this suggestion. As far as the importance of polysaccharides in the chemical structure of cuticle has been recently highlighted [74–76], it is likely that the observed differences in the barrier properties of the two types of cuticular isolates may be caused by hydrolysis of the cuticular polysaccharides during the acid treatment. Furthermore, potential influence of the isolation methods on the wax compositions of cuticles cannot be discarded. Therefore, a comprehensive chemical assay of the isolated cuticles is still needed before a definite choice of the most appropriate isolation procedure. Alternatively, a compromise between the two methods could be achieved by supplying the more user-friendly enzymatic method of isolation with a subsequent step of cuticle purification (e.g. treatment with chloroform or another organic solvent [19, 77].

Moreover, it was clearly demonstrated that the relevant information on the in situ barrier performance of the cuticle is given only when the astomatous cuticular membranes are used. In the case of stomatous membranes, no information is obtained about the stomatal penetration pathway, because the guard cells, which control opening and closing of the stomata in the intact plant, are lost during the isolation process. As far as several authors have stressed the general importance of this entry route [78, 79], stomatal absorption of humic substances remains to be an important issue for the future experimental concern. Nevertheless, since the liquid foliar formulations are usually primarily supplied to astomatous adaxial sides of leaves, we consider the model of transcuticular diffusion using this type of isolated membrane reasonable.

It is worth highlighting that the experimental arrangement of the diffusion experiment (diffusion couple with almost constant concentration of the solute in the donor compartment) has no ambition to simulate the real conditions during the foliar feeding process, where the solute concentration is changing dramatically in time by the evaporation, washing out by rain etc. Its application is aimed to answer the specific research questions concerning penetration of humic-based substances into leaves, such as the characterization and comparison of permeability of cuticles of different species to the single tested humate or the barrier properties of a specific cuticle against humic-based solutes of different size or solubility. It provides information on an upper limit of the rate of diffusional transport through cuticle. As was illustrated on the presented data, the barrier properties can be easily quantified. In this work, we used just the temporal development of the total diffusion flux for the quantification purposes. Nevertheless, if some additional experimental parameters were provided (e.g. the equilibrium amount of solute absorbed by the receptor compartment), it would be possible to calculate also further quantitative transport parameters, such as permeances or diffusion coefficients. The mathematical apparatus for these calculations are well described [71, 80]. On the basis of these parameters, barrier performance of cuticles against humic substances and other solutes (nutrients etc.) can be directly compared. Moreover, this applies also to comparison of the barrier properties of different types of membranes; permeability of a specific humate through a cuticle can hence be compared to the values obtained for different synthetic membranes etc.

## Conclusions

The results of the performed diffusion experiments revealed usability of described methodology for the study on the transcuticular transport of humic-based biostimulants. The proposed methodology represents a simple and cheap experimental tool. Nevertheless, the penetration experiments provide only one part of the overall perspective on all processes which take place in a collective manner when the humate penetrates the cuticle from the liquid product into the leaf. One crucial separate process which deserves more detailed description is the adsorption of the humate on the cuticle surface. The assessment of such parameters as the total adsorption capacity and sorption isotherm, or an explanation of the sorption mechanism and kinetics would lead to a better understanding the specific effects and modes of operation of humate-based biostimulants after their application on plants.

## Data Availability

The datasets used and/or analysed during the current study are available from the corresponding author on reasonable request.

## Abbreviations

PMMA: polymethylmethacrylate
UV–VIS: ultraviolet and visible spectroscopy
